# The Regulatory Effects of Paeoniflorin and Its Derivative Paeoniflorin-6′-O-Benzene Sulfonate CP-25 on Inflammation and Immune Diseases

**DOI:** 10.3389/fphar.2019.00057

**Published:** 2019-02-05

**Authors:** Jiajie Tu, Yawei Guo, Wenming Hong, Yilong Fang, Dafei Han, Pengying Zhang, Xinming Wang, Heinrich Körner, Wei Wei

**Affiliations:** ^1^Key Laboratory of Anti-Inflammatory and Immune Medicine, Ministry of Education, Anhui Collaborative Innovation Center of Anti-inflammatory and Immune Medicine, Institute of Clinical Pharmacology, Anhui Medical University, Hefei, China; ^2^The First Affiliated Hospital of Anhui Medical University, Hefei, China

**Keywords:** Pae, CP-25, inflammation, arthritis, treatment

## Abstract

The plant extract “total glucosides of peony” (TGP) constitutes a mixture of glycosides that is isolated from the roots of the well-known traditional Chinese herb *Paeonia lactiflora Pall*. Paeoniflorin (Pae) is the most abundant component and the main biologically active ingredient of TGP. Pharmacologically, Pae exhibits powerful anti-inflammatory and immune regulatory effects in some animal models of autoimmune diseases including Rheumatoid Arthritis (RA) and Systemic Lupus Erythematosus (SLE). Recently, we modified Pae with an addition of benzene sulfonate to achieve better bioavailability and higher anti-inflammatory immune regulatory effects. This review summarizes the pharmacological activities of Pae and the novel anti-inflammatory and immunomodulatory agent Paeoniflorin-6′-O-benzenesulfonate (CP-25) in various chronic inflammatory and autoimmune disorders. The regulatory effects of Pae and CP-25 make them promising agents for other related diseases, which require extensive investigation in the future.

## Introduction

### The Developmental Process of TGP-Pae-CP-25

The glycoside mixture TGP is an active plant extract that is isolated from the roots of *Paeonia lactiflora Pall*, a traditional Chinese medicine (TCM). In 1998, TGP was approved by the National Medical Products Administration (NMPA) for anti-inflammatory and immunomodulatory therapy in China. It has been used in the treatment of Rheumatoid Arthritis (RA) and Systemic Lupus Erythematosus (SLE) (Chen et al., 2013; Wang et al., 2014). Meanwhile a series of further studies has demonstrated that TGP also has therapeutic value in the treatment of chronic nephritis (Zhang et al., 2014), atherosclerosis (Li et al., 2011), and Sjögren’s syndrome (Li et al., 2013). Although TGP is effective without toxicity or off-target effects being evident, it has been shown to have a slow onset time by oral administration (Wang C. et al., 2012).

The main ingredients of TGP include Paeoniflorin (Pae), hydroxy-paeoniflorin, albiflorin, and benzoylpaeoniflorin (Absolute et al., 1972). Among them, Pae is the most abundant component (> 40%) and the main biologically active ingredient (Su et al., 2010). Recent investigations of Pae exhibited anti-inflammatory (Chang et al., 2011; Wang et al., 2013), anti-neoplastic (Sun, 2013), anti-hyperglycemia (Yang et al., 2004), and neuroprotective effects (Liu et al., 2005). Since Pae is characterized as a monoterpene, water-soluble glucoside with a low lipophilicity, bioavailability of Pae is relatively low due to insufficient absorption across the gastrointestinal epithelium after oral administration (Takeda et al., 1995). Extensive studies have been conducted to improve the bioavailability of Pae. Two well-known P-glycoprotein (P-gp) inhibitors, verapamil, and quinidine, can significantly elevate the absorption of Pae (Chan et al., 2006; Chen Y. et al., 2012). Furthermore, modification of acetylation improves the absorption and lipophilicity of Pae *in vitro* (Yang et al., 2013) and the bioavailability of benzoylpaeoniflorin sulfonate was increased and tested in a mouse model (Cheng et al., 2010). Based on this principle, we prepared paeoniflorin-6′-O-benzene sulfonate (CP-25). The oral bioavailability of CP-25 was much better than Pae in rats, and its anti-inflammatory and immunoregulatory effects were also significantly higher than Pae and TGP.

**FIGURE 1:**
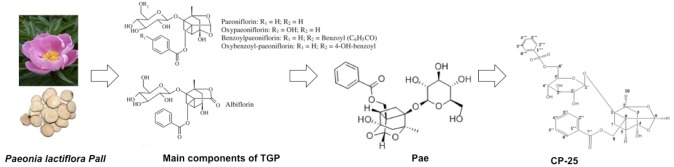
The developmental process of TGP-Pae-CP-25.

In this review, we summarize the recent progress in the use of Pae in immunoregulatory and anti-inflammatory activities, including the regulation of RA, kidney/liver injury and other immune-related diseases. In addition, we also review the pharmaceutical effects of CP-25 on inflammation and immune-associated diseases to highlight the use of Pae and its derivative CP-25 as potential agents for subsequent research and clinical application (Figure 1).

## The Pharmacological Effect of Pae in Inflammatory Pathologies

### Arthritis

The anti-inflammatory and immunoregulatory effects of Pae on mesenteric lymph node (MLN) lymphocytes and the underlying mechanisms were investigated in an adjuvant arthritis (AA) rat model. Pae greatly reduced arthritis scores and secondary hind paw swelling, pro-inflammatory cytokine production and the proliferation of MLN lymphocytes. Pae induced the expression of β2-adrenergic receptor (ADRB2) and decreased that of β-arrestin1/2 in MLN lymphocytes. In addition, Pae reversed the pro-inflammatory cAMP of MLN lymphocytes *in vitro*. The effects of Pae on ADRB2 desensitization and β2-cAMP signal transduction in MLN lymphocytes is essential for arthritis pathogenesis (Wu et al., 2007). In another publication using AA rat model, Pae repressed the malondialdehyde and induced superoxide dismutase, catalase, and glutathione peroxidase in blood. Moreover, Pae inhibited nuclear factor-κB (NF-κB) p65 unit, tumor necrosis factor (TNF)-α, interleukin (IL)-1β and IL-6, and reduced the COX-2 (Jia and He, 2016). These reports suggested that Pae ameliorates disease of AA rat.

Zhai et al. (2018) showed that Pae relieved Collagen-Induced Arthritis (CIA) rats by repressing Rho kinase (ROCK), p-NF-κB p65 and inflammatory cytokines, such as TNF- α and IL-6, in the joint synovial tissues. Wu et al. (2013) also showed that the expressions of inflammatory cytokines were repressed in Pae-treated CIA rat. Additionally, Pae could repress TNF- α and IL-1β in serum and ameliorate bone erosion of CIA rat. Inflammatory mediators and G protein-coupled signaling were associated with the pathogenesis of synovitis in CIA rats that was attenuated by Pae. Our group demonstrated that Pae could inhibit hyperpalsy and inflammatory cytokine production of fibroflast-like synoviocytes (FLS) from CIA via inhibiting Gi expression and restoring cAMP level and PKA activity (Zhang et al., 2008).

GRK2 and β-arrestin1/2 also belong to G-protein-coupled receptors (GPCRs) signaling family. GRK2 increases in synovium from CIA rats and GRK2 inhibitor suppresses proliferation and induces the cAMP/PKA activity of FLS. Moreover, Pae shows similar effects in the CIA model via inhibiting GRK2 expression in FLS which mirrors the effects of a GRK2 inhibitor. These results suggest that Pae could ameliorate the inflammatory status in CIA via regulating GRK2 in FLS (Chen J.-Y. et al., 2012). To further explore the effect of Pae on β-arrestin 2 in humans, FLS were isolated and cultured *in vitro*. It was shown that IL-1β-induced β-arrestin 2 suppresses the cAMP-PKA signaling pathway and promotes FLS proliferation. Addition of Pae inhibits FLS proliferation and up-regulates expression of β-arrestin 2 in human FLS. This implies that Pae could repress IL-1β-induced human FLS proliferation via modulation of β-arrestin 2-cAMP-PKA pathway (Wu et al., 2012).

Besides inhibition of FLS proliferation and suppression of inflammatory cytokines through activating the E-prostanoid (EP4) receptor protein expression and modulating intracellular cAMP level, Pae also inhibited thymocyte and splenocyte proliferation in CIA rats (Chang et al., 2011). PI3K/Akt/mTOR signaling mediated by BAFF/BAFF-R participates in antibody production by B lymphocytes of CIA rats. Pae had therapeutic effects on CIA rats via regulating PI3K/Akt/mTOR signal mediated by B cell-activating factor belonging to the TNF family (BAFF)/BAFF-R. Thus Pae could repress antibodies production from B cells (Li et al., 2012). Taken together, Pae exerts powerful anti-inflammatory effects via mainly modulating PEG2-β-arrestin 2-cAMP-PKA in various immune cells, such as B cells, FLS, thymocytes, and splenocytes. Taken together, these results from AA and CIA rat models laid the foundation for further study of Pae in RA therapy.

Osteoarthritis (OA) is another main type of arthritis. Pae treatment also showed therapeutic effects on OA chondrocytes. Pae repressed IL-1β-induced inflammatory factors, including NO, PGE2, iNOS and COX-2, in chondrocytes from OA patients. Moreover, Pae repressed the IL-1β-induced metalloproteinase-3 (MMP-3), MMP-13 and NF-κB p65 in OA patient-derived chondrocytes. Therefore Pae may ameliorate IL-1β-stimulated infammatory factors in chondrocytes from OA patients by inhibiting the activation of the NF-κB pathway (Zhao L. et al., 2018). What’s more, Pae repressed the production of lactate dehydrogenase (LDH) and IL-1β-stimulated apoptosis of rat chondrocytes via activating Akt signaling pathway (Kogut et al., 2005). To summarize, Pae may exert its therapeutical effect by inhibiting harmful effects of chondrocytes and thus, may be a potential agent in the future treatment of OA.

### Liver Diseases

Pae treatment showed protective effect for several liver diseases. Ma et al. (2016) demonstrated that the Pae repressed serum alanine transferase (ALT), aspartate transferase (AST) and total levels of cholesterol (TC), low-density lipoprotein (LDL), and TNF-α, from a non-alcoholic steatohepatitis (NASH) rat model via inhibiting Rho kinase (ROCK) and NF-κB pathway. Zhao et al. (Zhao et al., 2017) evaluated the effect of Pae on α-naphthylisothiocyanate (ANIT)-induced cholestasis rat model. Pae inhibited neutrophils infiltration, edema and necrosis in liver tissue. Additionally, Pae repressed the as total bilirubin (TBIL), direct bilirubin (DBIL), AST, ALT, alkaline phosphatase (ALP), γ-glutamyltranspeptidase (γ-GT), total bile acid (TBA) from serum samples that isolated from ANIT-treated rats. The liver expression of NF-κB and IL-1β were repressed and the hepatocyte transporters such as Na+/taurocholate cotransporting polypeptide (NTCP), bile salt export pump (BSEP), multidrug resistance-associated protein 2 (MRP2) were reduced by Pae treatment. The alleviating effect of Pae on the liver seems to be closely associated with down-regulation of activated NF-κB pathway.

The elevated IL-8 is positively related to inflammatory liver diseases, implying that IL-8 inhibition may be a potential treatment of inflammatory liver diseases. Pae ameliorated IL-8-induced liver damage by exerting anti-inflammatory effects on primary human hepatic sinusoidal endothelial cells (HHSECs) through inhibiting IL-8 via repression of ERK1/2 and Akt pathway (Gong et al., 2015). Pae pretreatment reduced the elevated plasma aminotransferase expression and liver necrosis in Concanavalin A (Con A)-induced hepatitis mice model. Moreover, Pae pretreatment repressed the proinflammatory cytokines and infiltration of CD4^+^, CD8^+^ and NKT cells in liver. Pae pretreatment also could inhibit the Toll-like receptor (TLR) 4 and NF-κB pathway in Con A-induced liver. These results suggested that Pae pretreatment protects mice against Con A-induced liver damage via suppression of several inflammatory factors and infiltration of CD4^+^, CD8^+^ and NKT cells in liver, and Pae might exert this therapeutical effect through inhibition of TLR4 and NF-κB pathway (Chen et al., 2014).

Hepatic ischemia/reperfusion (I/R) injury could induce several side effects and even death after liver resection and transplantation. Pae treatment significantly inhibited I/R-induced serum ALT and AST, hepatic damages/apoptosis, and neutrophils infiltration in liver. The secretion of pro-inflammatory cytokines and expression of high mobility group box-1 (HMGB1), TLR4, phosphorylated ERK1/2, JNK1/2, p38, and NF-κB was repressed by Pae treatment in the I/R-operated mice, suggesting that that Pae also could protect liver I/R injury via suppressing HMGB1-TLR4 pathway (Xie et al., 2018).

Zhang et al. (2015) evaluated the effects of Pae on Nonalcoholic fatty liver disease (NAFLD) by using a high-fat diet mice model. Pae prevented NAFLD development by down-regulating inflammation (phosphoenolpyruvate carboxykinase and G6Pase), hyperlipidemia (lipid synthesis pathway [3-hydroxy-3-methyl glutaryl coenzyme A reductase (HMG-CoAR) and peroxisome proliferator-activated receptor (PPAR) pathways], insulin resistance and body weight. In addtion, Pae also protected the cardiovascular system of NAFLD mice. Kim et al. investigated the protective effects of Pae on lipopolysaccharide (LPS)-induced inflammation in rat liver. Pae pretreatment repressed LPS-induced glutamate oxaloacetate transaminase, lactate dehydrogenase, glutamate pyruvate transaminase, and malondialdehyde. Additionally, Pae treatment resotred LPS-repressed superoxide dismutase, glutathione peroxidase, and catalase. Pae protected LPS-stimulated damage of liver tissue, suggesting that Pae indeed could ameliorate LPS-induced liver inflammation (Kim and Ha, 2010).

In addition, a potentially important feature of Pae treatment is its ability to protect against hyperplasia of hepatic stellate cells (HSCs) and significant depositions of collagen type I (Col I) and type III (Col III) in experimental schistosomiasis by modulation of TNF-α, IL-6, lipopolysaccharide binding protein (LBP) and CD14 expressions (Liu et al., 2006).

Murine peritoneal macrophages secrete Transforming growth factor beta (TGF-β) 1 after activation with Soluble Egg Antigen (SEA) of *Schistosoma japonicum.* This causes HSCs proliferation and secretion of Col I and III. Addition of Pae to macrophage-conditioned medium inhibits these pathological features of hepatic fibrosis HSCs (Chu et al., 2007). IL-13 is closely associated with the development of schistosome fibrosis. While IL-13 receptor (R) a2 is an effective target in attenuation of fibrosis. A mouse model for liver fibrosis was established by subcutaneous infection with *S. japonicum cercariae*. Pae had suppressive effect on the increase of both hepatic hydroxyproline and Col I and III, which are the main components of extracellular matrix (ECM). Moreover, Pae inhibited IL-13 production and elevates IL-13Ra2 in Pae-treated groups. Therefore Pae meliorated liver fibrosis via rebalancing of IL-13 and IL-13Ra2 (Li et al., 2009). In another IL-13 related study, Pae inhibited IL-13-induced collagen synthesis in the *in vitro* culture of primary hepatic stellate cells (HSCs), implying that Pae could alleviate the hepatic granulomas and fibrosis via modulating IL-13 signaling pathway in HSCs (Li et al., 2010). Moreover, IL-13 secretion was up-regulated from liver alternative activated macrophages. Pae repressed Signal transducer and activator of transcription (STAT) 6, phosphorylations of janus-activated kinase 2 (JAK2), and Arginase-1 in alternative activation of macrophages, then causing repression of IL-13 secretion. Therefore, Pae is a promising prophylactic agent for hepatic granuloma and fibrosis of schistosomiasis japonica (Chu et al., 2011).

Prostaglandin E2 (PGE2) and its four prostanoid receptors (EP1-4) are involved in tumor development and progression (Aoki and Narumiya, 2017). Pae significantly inhibited the proliferation and induced apoptosis in butaprost-stimulated HepG2 and SMMC-7721 cells. Pae induced apoptosis in hepatocellular carcinoma cells by moudulating PGE2-EP2 pathway and inducing the Bax-to-Bcl-2 ratio, suggesting that Pae might be a promising agent in the treatment of liver cancer (Hu et al., 2013).

### Kidney Diseases

High glucose activated macrophages mainly through TLR2-dependent pathway which aggravated the severity of renal inflammation and eventually contributed to diabetic nephropathy (DN). Pae might be used as a potential therapeutic agent against progressive DN (Shao et al., 2016). *In vivo*, Pae reduced the urinary albumin excretion rate and inhibit macrophage infiltration and activation through inhibition of the TLR2/4 pathway. *In vitro*, Pae reduced the advanced glycation end products (AGEs)-induced TLR2/4 activation and inflammatory responses. These findings indicated that Pae prevents macrophage activation via regulating TLR2/4 signaling activation in DN (Zhang T. et al., 2017). The effects of Pae on the kidneys of mice with streptozotocin-induced type 1 diabetes mellitus was evaluated by using TLR2 knockout mice (TLR2-/-). After 12 weeks of Pae treatment, diabetic mice had significantly reduced albuminuria and attenuated renal histopathology. These changes were associated with substantially alleviated macrophage infiltration and down-regulation of TLR2 signaling pathway. These data supported the idea that the curative effects of Pae on the kidney of diabetic mice are associated with the regulation of the TLR2 pathway. Pae thus shows therapeutic potential for the prevention and treatment of DN (Shao et al., 2017).

Zhang et al. (2013) evaluated the protective activities of Pae on advanced glycation end product-induced oxidative stress and inflammation in mesangial cells. Pae pretreatment induced advanced glycation end product-induced glutathione peroxidase and catalase and repressed the macrophages migration in a co-culture system of mesangial cells and macrophages. In addition, the advanced glycation end products-induced IL-6 and monocyte chemoattractant protein (MCP)-1 was repressed by Pae pretreatment. These results showed that Pae could ameliorate advanced glycation end products-induced oxidative damage and inflammation in mesangial cells.

The potential treatment effects of Pae on acute renal injury induced by acute necrotizing pancreatitis (ANP) were investigated in a rat model. Pae repressed acute renal injury by suppressing inflammatory reaction and apoptosis of renal cell via modulaing p38MAPK and NF-κB pathway (Wang et al., 2016). Another paper (Liu et al., 2016) reported the protecetive effects of Pae on renal function by using a cyclophosphamide (CYP)-induced mice model. Pae ameliorated the damage of kidney tissues, such as apoptosis, caused by CYP. Pae decreased the uric acid and creatinine in urine and inflammatory cytokines in serum through up-regulating AMPK pathway and down-regulating NF-κB pathway. Therefore Pae is a potential agent for kidney toxicity.

## Other Inflammatory Related Conditions

Recombinant human interleukin-1b (rhIL-1β) was used to treat primary monocytes to imitate inflammatory condition *in vitro*. Pae showed low cytotoxicity on rhIL-1β-treated monocytes. Pae significantly suppressed phagocytic function of rhIL-1β-induced monocytes, and decreased the levels of TNF-α and PGE2 production. Administration of Pae significantly inhibited the HLA-DR and CD80 with rhIL-1β-stimulated monocytes. These results indicated that Pae could inhibit activation and normal function of monocytes in human peripheral blood (Wang D. et al., 2012).

Another similar study focused on the effect of Pae on rhIL-1β-stimulated human peripheral blood mononuclear cells (PBMCs). Pae inhibited the proliferation of rhIL-1β-treated PBMCs and production of IL-17 and IL-10. rhIL-1β-induced down-regulation of PBMCs CD4^+^CD25^+^Foxp3^+^ subpopulation numbers was also repressed by Pae. Therefore, Pae exerts its anti-inflammatory effects via regulating IL-17/IL-10 secretion (Dai et al., 2015).

Topical application of dinitrochlorobenzene (DNCB) induced cutaneous inflammation. Thymocyte proliferation in the mice with allergic contact dermatitis (ACD) was significantly inhibited by Pae. IL-4/IL-10 production was induced and IL-2/IL-17 was redued in Pae-treated thymocyte and splenocyte. Therefore anti-inflammatory action of Pae in the murine model of allergic ACD is through its regulation of the imbalanced cytokine production (Wang et al., 2013). In summary, the anti-inflammaotry and immune regulatroy roles of Pae was summarized in Table 1.

**Table 1:** The summary of curative effects of Pae.

Disorder’s and related models		Related cells	Related target of pathway	Reference
Arthritis	AA rats	Mesenteric lymph node (MLN) lymphocytes	B2-AR and β-arrestinl/2-cAMP	Wu et al., 2007
		Blood sample and joint tissue	NF-kB pathway	Jia and He, 2016
	CIA rats	FLS	Gi-cAMP-PKA	Zhang et al., 2008
			GRK2	Chen Y. et al., 2012
		Thymocyte and sptenocyte	EP4-CAMP	Chang et al., 2011
		B lymphocytes	TNF family (BAFF)/BAFF-R-P13K/Akt/mTOR	Li et al., 2012
		Joint synovium	Rho kinase, NF-KB pathway	Zhai et al., 2018
		Serum	TNF-a, IL-1β	Wu et al., 2013
	OA	Human chondrocyte	NF-KB pathway	Zhao M. et al., 2018
		Rat chondrocyte	Akt pathway	Kogut et al., 2005
	IL-1β-induced inflammation	Human FLS	β-arrestin 2-cAMP-PKA	Wu et al., 2012
Liver disease	Immunological liver injury	Mice liver	TNF-a, IL-6, LBP and CD14	Liu et al., 2006
	Non-alcoholic steatohepatitis	Rat liver	ROCK/NF-KB pathway	Ma et al., 2016
	Cholestasis	Rat liver	NF-KB pathway and hepatocyte transporters	Zhao et al., 2017
	IL-8 induced inflammatory damage	Primary human hepatic sinusoidal endothelial cells	ERK1/2 and Akt pathway	Gong et al., 2015
	Con A-induced hepatitis	Mice liver	TLR4 and NF-KB pathway	Chen et al., 2014
	Hepatic ischemia/reperfusion injury	Mice liver	HMGB1-TLR4 pathway	Xie et al., 2018
	Nonalcoholic fatty liver disease	Rat liver	PPAR pathway	Zhang et al., 2015
	LPS-induced liver inflammation	Rat liver	Oxidative stress markers	Kim and Ha, 2010
	Fibrosis	Macrophages conditional medium-treated HSCs	TGF-β1 signaling	Chu et al., 2007
		Mice liver	IL-13 and IL-13Ra2	Li et al., 2009
		Hepatic stellate cells	IL-13 pathway	Li et al., 2010
	Hepatic granuloma	Macrophages	JAK-STAT pathway	Chu et al., 2011
	Liver cancer	HepG2 and SMMC-7721 cells	PGE2-EP4	Hu et al., 2013
Kidney disease	Diabetic nephropathy (ON)	Macrophage	TLR2/4 signaling	Shao et al., 2016; Zhang T. et al., 2017; Shao et al., 2017
	Advanced glycation end product-induced oxidative stress and inflammation in mesangial cells	Mesangial cells and macrophages	None	Zhang et al., 2013
	Acute renal injury	Kidney tissue	MAPK and NF-KB pathway	Wang et al., 2016
	Cyclophosphamide - induced renal damage	Kidney tissue	MAPK and NF-KB pathway	Liu et al., 2016
Other disorders	rhIL-16-induced inflammation	Circulating Monocyte	HLA-DR and CD80	Wang D. et al., 2012
		PBMC	1L-17 and 1L-10	Dai et al., 2015
	Allergic contact dermatitis	Thymocyte and splenocyte	IL-4/IL-10andlL-2/IL-17	Wang et al., 2013


### Pharmacokinetics (PK)

The pharmacokinetics of Pae microemulsion and Pae saline was compared by our group. Pae microemulsion and Pae were given to AA rats for 10 days. Compared to the Pae group, the area under the plasma concentration-time curve [AUC(0-t)], maximum concentration [C(max)] and mean retention time MRT(0-infinity))(h) of Pae microemulsion were up-regulated, while volume of distribution (Vd) and clearance rate (CL/F) decreased, suggesting that microemulsion significantly improves the absorption of Pae in AA rats (Wang C. et al., 2012).

## The Pharmacological Effects of CP-25

### Arthritis

By using a T cell and FLS co-culture system, CP-25 repressed the proliferation and production of pro-inflammatory cytokines of FLS via inhibiting BAFF-R in CD4^+^ T cells, suggesting that CP-25 could interfere in the crosstalk between T cells and FLS *in vitro* (Jia et al., 2016). The AA model was used to investigate the anti-arthritic activity of CP-25. In general, CP-25 repressed both the clinical and the histopathological scores of arthritis. The levels of pro-inflammatory cytokines, including IL-1β, IL-6 and TNF-α, were decreased and after CP-25 treatment the anti-inflammatory cytokine TGF-β1 could be detected in serum. Furthermore, CP-25 treatment polarized peritoneal macrophages from a M1 to a M2 phenotype, inhibited Th17-IL-17, suppressed the Th17-associated transcription factor RAR-related orphan receptor gamma (ROR-γt), the receptor activator of nuclear factor kappa B ligand (RANKL) and matrix metalloproteinase (MMP) 9 in AA rats (Chang et al., 2016).

### Other Chronic Inflammatory Diseases

Bone marrow dendritic cells (DCs) were isolated from BALB/c mice and stimulated by PGE2 and TNF-α, respectively, which induced CD40, CD80, CD83, CD86, and MHC-II and suppressed the antigen uptake by DCs. Additionally, the proliferation of T cells was induced using a co-culture system. The expression of surface markers, DC antigen uptake and DC-mediated proliferation of T cells were inhibited by CP-25 treatment. Moreover, CP-25 decreased PGE2-induced EP4 and NF-κB and induced PGE2-suppressed increase of cAMP in DCs. TNF-α-induced TNFR1, TRADD, TRAF2, and NF-κB were also inhibited by CP-25 in DC, suggesting that CP-25 modulates DCs immune function via regulating PGE2-EP4-cAMP and TNF-α-TNFR1-TRADD-TRAF2-NF-κB pathways (Li et al., 2015). While BAFF or TNF-α could induce B lymphocytes proliferation *in vitro* additional CP-25 treatment suppressed B lymphocytes proliferation. Moreover, CP-25 also reduced the numbers of B lymphocytes subtypes, including CD19^+^ B lymphocytes, CD19^+^CD20^+^ B lymphocytes, CD19^+^CD27^+^ B lymphocytes and CD19^+^CD20^+^CD27^+^ B lymphocytes, and down-regulated BAFF or TNF-α-induced expression of BAFFR, BCMA, and TACI. Interestingly, this study also compared the effects between Rituximab, Etanercept and CP-25 treatments. Results showed that addition of CP-25 moderately restored a hyper-activated B lymphocyte function to a physiological level by regulating the classical and alternative NF-κB signaling pathway mediated by BAFF. On the contrary, the inhibitory effects on BAFF-BAFFR-NF-κB pathway are much more obvious in Rituximab and Etanercept treatment groups. Therefore CP-25 is a promising anti-inflammatory immune agent as a modifying drug that minimize the potential side effects (Zhang F. et al., 2017).

A hallmark in patients with autoimmune diseases is the elevated presence of immunoglobulin D (IgD) (Wu et al., 2016, 2017). IgD binds to IgD receptor (IgDR) on CD4^+^ T cells from human PBMC, which leads to activation/proliferation of T cells by enhancing phosphorylation of the activating tyrosine residue of Lck (Tyr394). CP-25 treatment could repress the IgD-induced activation/proliferation of CD4^+^ T cells by repressing of Lck (Tyr 394) phosphorylation. These results demonstrate CP-25 is a novel potential therapeutic agent for human autoimmune diseases via modulating T cells function (Wu et al., 2018).

Primary Sjögren’s syndrome (pSS) is a chronic inflammatory autoimmune disease that is featured by various immune abnormalities in moisture-producing glands. CP-25 alimeorated the clinical manifestations, including salivary flow and histopathological scores, of in NOD/Ltj mice (a mice model of pSS). Compared to control group, lymphocyte viability and the infiltration of Th1/Th2 cells in salivary glands were repressed in CP-25-treated NOD/Ltj mice. Moreover, CP-25 treatment skewed the ratio of Th17/regulatory T (Treg) cells in the spleen on NOD/Ltj mice. A concentration of inflammatory cytokines and anti-La/SSB and IgG antibodies in the serum from NOD/Ltj mice was repressed by CP-25. This study suggested that CP-25 is a potential agent for pSS by regulating T lymphocyte subsets (Fang et al., 2018). Taken together, CP-25 was developed which enhanced lipophilicity and strong anti-inflammatory and immune regulatory properties (Table 2).

**Table 2:** The summary of curative effects of CP-25.

Disorders and related models		Related cells	Related target or pathway	Reference
Arthritis	AA rats	Macrophages	Thl7-IL-17, ROR-yt, RANKL	Kim and Ha, 2010
	CIA mice	T lymphocytes and FLS	β2-AR pathway	Liu et al., 2006
Other disorders	BAFF-induced inflammation	T lymphocytes and FLS	BAFF-R pathway	Chu et al., 2007
	PGE2 or TNF-α induced inflammation	DC and T lymphocytes	PGE2-EP4-cAMP and TNF-α-TNFR1-TRADD-TRAF2-NF-KB pathways	Li et al., 2009
	BAFF or TNF-α could induce inflammation	B lymphocytes	BAFF-BAFFR-NF-KB pathway	Li et al., 2010
	IgD-induced T cells activation	T lymphocytes	Lck (Tyr 394)	Chu et al., 2011
	Sjögren’s syndrome	Thl7/regulatory T (Treg) cells	Anti-SSB/La and IgG antibody	Aoki and Narumiya, 2017


### Absorption and Excretion

As a bioavailable derivative of Pae, CP-25 improves the absorption of Pae. This has been attributed to both the lipid solubility enhancement and its resistance to P-gp-mediated efflux (Yang et al., 2016). With regard to tissue distribution, CP-25 concentration was higher in most tissues when compared to Pae via an oral route with the highest concentration in the liver at 3 h after oral treatment. Other tissues, including intestine, synovium, muscle, lung, and brain in both male and female rat, also showed high concentration of CP-25. Following a single oral dose of 50 mg/kg in rats, CP-25 was primarily excreted in the feces. There is a gender-related difference in the tissue distribution and excretion (Zhao M. et al., 2018).

## Outlook

The mixture of glycosides Pae has been shown to be anti-inflammatory, anti-neoplastic, anti-hyperglycemia, and neuroprotective. To improve oral bioavailability of Pae. Paeoniflorin-6′-O-benzene sulfonate (CP-25) was developed which enhanced lipophilicity and significant increases could be shown in animal experiments. Current basic and preclinical studies imply that these natural compounds with their strong anti-inflammatory properties should be considered in the clinic. As shown in various preclinical studies, Pae and its derivative CP-25 demonstrated positive effects on chronic inflammatory diseases, such as arthritis, and in different models of liver injury and kidney injury. The use in therapy modulated inflammatory mediators such as cytokines (IL-1β, IL-6, TNF-α), chemokines (CCL8), pattern recognition receptors and their relevant transcription factors (STAT, NF-κB). Although the effectiveness of Pae and CP-25 has been demonstrated, aspects such as pharmacokinetics and product safety need to be considered. While CIA or AA rat models have been used widely the joint inflammation model K/BxN which is achieved by serum transfer would be a more reliable model to investigate the pathological mechanism of RA.

## Author Contributions

JT drafted the manuscript. YG, WH, YF, DH, PZ, XW, HK, and WW revised the manuscript.

## Conflict of Interest Statement

The authors declare that the research was conducted in the absence of any commercial or financial relationships that could be construed as a potential conflict of interest.
